# Geospatial analyses to identify clusters of adverse antenatal factors for targeted interventions

**DOI:** 10.1186/1476-072X-12-46

**Published:** 2013-10-24

**Authors:** Shanley Chong, Michael Nelson, Roy Byun, Liz Harris, John Eastwood, Bin Jalaludin

**Affiliations:** 1 Centre for Research, Evidence Management and Surveillance, South Western Sydney Local Health Districts, Sydney, Australia; 2 South Western Sydney Clinical School, University of New South Wales, Sydney, Australia; 3 NSW Biostatistical Officer Training Program, NSW Ministry of Health, Sydney, Australia; 4 Research Centre for Primary Health Care and Equity, University of New South Wales, Sydney, Australia; 5 South Western Sydney Local Area Health District, Sydney, Australia; 6 School of Public Health and Community Medicine, University of New South Wales, Sydney, Australia; 7 School of Women’s and Children’s Health, University of New South Wales, Sydney, Australia; 8 School of Public Health, University of Sydney, Sydney, Australia

**Keywords:** Antenatal, Pregnancy, Smoking, SaTScan, Geospatial

## Abstract

**Background:**

Late antenatal care and smoking during pregnancy are two important factors that are amenable to intervention. Despite the adverse health impacts of smoking during pregnancy and the health benefits of early first antenatal visit on both the mother and the unborn child, substantial proportions of women still smoke during pregnancy or have their first antenatal visit after 10 weeks gestation. This study was undertaken to assess the usefulness of geospatial methods in identifying communities at high risk of smoking during pregnancy and timing of the first antenatal visit, for which targeted interventions may be warranted, and more importantly, feasible.

**Methods:**

The Perinatal Data Collection, from 1999 to 2008 for south-western Sydney, were obtained from the New South Wales Ministry of Health. Maternal addresses at the time of delivery were georeferenced. A spatial scan statistic implemented in SaTScan was then used to identify statistically significant spatial clusters of women who smoked during pregnancy or women whose first antenatal care visit occurred at or after 10 weeks of pregnancy.

**Results:**

Four spatial clusters of maternal smoking during pregnancy and four spatial clusters of first antenatal visit occurring at or after 10 weeks were identified in our analyses. In the maternal smoking during pregnancy clusters, higher proportions of mothers, were aged less than 35 years, had their first antenatal visit at or after 10 weeks and a lower proportion of mothers were primiparous. For the clusters of increased risk of late first antenatal visit at or after 10 weeks of gestation, a higher proportion of mothers lived in the most disadvantaged areas and a lower proportion of mothers were primiparous.

**Conclusion:**

The application of spatial analyses provides a means to identify spatial clusters of antenatal risk factors and to investigate the associated socio-demographic characteristics of the clusters.

## Introduction

There are many established risk factors for adverse perinatal outcomes. These include late antenatal care, smoking during pregnancy, maternal infection [1], maternal hypertension [2], gestational diabetes [3] and social factors such as teen pregnancy [4] and lower socioeconomic status [5]. Late antenatal care and smoking during pregnancy are two important factors that are amenable to intervention.

Australia’s and UK’s NICE antenatal care guidelines recommend that the first antenatal visit should be before 10 weeks of gestation [6,7]. Early first prenatal care is important for the detection of adverse pregnancy related outcomes and is vital for healthy perinatal outcomes in both mothers and infants [8-10]. The benefits include healthy birth weight [11-13], low risk of preterm delivery [12,14] and lower neonatal mortality [12,14]. Antenatal care programs not only monitor both maternal and foetal health, but also facilitate health promoting advice such as smoking cessation. Women of low socio-economic status [15,16], younger women [15], primiparous women [17,18] and indigenous women [19] are associated with late antenatal care.

Smoking during pregnancy is associated with a high risk of miscarriage, stillbirth and serious complications during delivery [20], all of which add to the overall health costs [21]. Younger women are more likely to smoke during pregnancy [22] and mothers of lower socio-economic status, who were primiparous and attended first antenatal care late were less likely to quit smoking during pregnancy [23].

Despite the negative health impacts of smoking during pregnancy and the health benefits of early first antenatal visit on both mother and unborn child, substantial proportions of women still smoke during pregnancy or have their first antenatal visit after 10 weeks gestation. For example, 20% of mothers who gave birth during 2002 to 2004 smoked during pregnancy in Western Australia [24] and in New South Wales (NSW), 41% of women had their first antenatal care late in their pregnancies [17]. Therefore, it is important for health care providers to ensure that both the care providers and the community are aware of the benefits of early prenatal care and the detrimental effects of smoking during pregnancy on maternal and infant health.

Although targeted preventative programs such as a home visiting program can improve maternal health-related behaviours during and after pregnancy [25,26], it can be difficult to direct interventions to individual mothers at greatest need. The identification of small geographical areas with a high prevalence of poor maternal-related behaviour could allow for more targeted efforts at those most at risk.

The application of spatial statistics and geographic information system (GIS) to health outcomes is now being increasingly used to provide novel ways of examining disease patterns geographically [27-29]. SaTScan is a software for implementing spatial, temporal and space-time scan statistics that can be used to determine areas where an event of interest, for example, cancer incidence or preterm deliveries, appears inconsistent with the overall study area. This technique is not limited to existing administrative boundaries such as postal area, and can identify location of the clusters without a priori knowledge about their location and size [30].

To our knowledge, SaTScan has not been used for analysing geographical patterns of antenatal maternal-related risk factors. This study was undertaken to assess the usefulness of such geospatial methods in identifying communities at high risk of poor maternal-related behaviours, particularly, smoking during pregnancy and timing of the first antenatal visit, for which targeted interventions may be warranted and importantly feasible.

## Methods

The study area was the south-western region of metropolitan Sydney, Australia. It is about 6382 square kilometres in area, consists of 15 local government areas (LGAs) and had a population of 1,460,847 in the 2011 Census [31]. The population in the 15 LGAs ranged from 32,423 to 187,766 persons, and the population density ranged from 17 to 6,349 persons per square kilometre. In the same census, Metropolitan Sydney covers about 12,368 square kilometres and had a population of 4,391,674 persons [31].

### Data source

The Perinatal Data Collection (MDC), from 1999 to 2008 for south-western Sydney, was obtained from the New South Wales (NSW) Ministry of Health. It is a population-based surveillance system comprising of all births in NSW public hospitals, private hospitals and homebirths. It includes all livebirths and still births of at least 20 weeks gestation or at least 400 grams birth weight. A notification form is completed by the attending midwife for every birth. Information is collected on demographic items and items on maternal health, pregnancy, labour, delivery and the newborn and includes maternal age, smoking during pregnancy (yes/no), timing of first antenatal visit, birth weight, gestational age, single or multiple births, parity and country of birth.

Using the Australian antenatal guidelines, timing of the first antenatal visit was categorised into two groups: first antenatal visit <10 weeks and first antenatal visit ≥10 weeks (late first antenatal visit) [6,7].

The 2001 and 2006 Index of Relative Socio-Economic Disadvantage (IRSED) at the census collection district (CCD) level were used in the analyses as an ecological measure of area deprivation [32]. A CCD consists of about 220 households in urban areas and fewer households in rural and semi-rural areas. The IRSED at the CCD level was created by the Australian Bureau of Statistics to compare social and economic disadvantage across geographical areas in Australia. The index is derived from Census variables such as low income and educational attainment, high unemployment, and people working in unskilled occupations. The index has a mean score of 1,000 and standard deviation of 100 [33]. The IRSED was categorised into quintiles. Quintile 1 is designated as most disadvantaged, quintile 2 to quintile 4 are combined and designated as the middle disadvantaged group and quintile 5 is designated as the least disadvantaged group. All women living in a particular CCD were assigned the IRSED for that CCD.

### Statistical methods

Maternal residential addresses at the time of delivery were georeferenced and imported into spatial scan statistic (SaTScan) for analysis. A spatial scan statistic implemented in SaTScan was used to identify the presence of statistically significant spatial clusters of women who smoked during pregnancy or women whose first antenatal care visit occurred ≥10 weeks of pregnancy [34]. The analysis was conducted using a Bernoulli model, binary event data. Women who did not smoke during pregnancy or whose first antenatal care occurring before 10 weeks of pregnancy were assigned as controls. A spatial scan statistic uses a scan window (the population at risk) either in the shape of a circle or an ellipse, which moves across the study region [34,35]. We present our results using the ellipse window and a medium strength compactness penalty because it provided higher sensitivity than the circular-shaped window for late antenatal visit (53% vs 49%, respectively) and similar sensitivity for maternal smoking (42% vs 43%, respectively).

For each location, the size of the scan window varies from 0 to a specified maximum value. For the purposes of this study, the size of the scan window was set to no more than 20% of the study population, to scan for small clusters which may possibly be more amenable to interventions. For each window, the alternative hypothesis is that there is a difference in the risk of poor maternal health behaviour within the window as opposed to outside the window. The likelihood function is maximised over all windows, and the window with the maximum likelihood constitutes the most likely cluster. We selected the non-overlapping option in SaTScan when generating secondary clusters. Clusters with significant large likelihood ratios are identified. The test of significance of the identified clusters is based on a likelihood ratio test whose p-value is generated by applying Monte Carlo replications [35,36]. The number of Monte Carlo replications was set to 999 to ensure adequate power for defining clusters and a p-value less than 0.05 was considered statistically significant.

Using purely spatial scan statistics, we also examined for clusters using two year time periods (1999–2000, 2001–2002, 2003–2004, 2005–2006, 2007–2008). The locations and sizes of the clusters did not vary greatly by these two periods. The spatial-temporal clusters that we detected were similar in locations and size to the spatial only clusters, except for late antenatal visit between 2007 and 2008 where two new clusters were found. These two clusters were also detected using purely spatial scan and space-time scan statistics. This indicates the occurrence of maternal smoking is mainly spatial rather than temporal in our study area. For simplicity, only spatial clusters based on purely spatial scan were presented.

The specific locations of clusters were evaluated in terms of relative risks (RRs). A cluster with a RR of >1 indicates an increased risk for that cluster compared to the risk outside that cluster. Kernel density was then used to visually explore the variability of the density over the surface of these clusters into Google map using R-studio [37]. To account for correlation among women within clusters, generalised estimating equations (GEE) logistic regression models with logit link and compound symmetry correlation were used to examine the associations between socio-demographic and clinical characteristics of women inside and outside the significant clusters.

Ethics approval for this study was obtained from the NSW Population & Health Services Research Ethics Committee.

## Results

**Figure 1:**
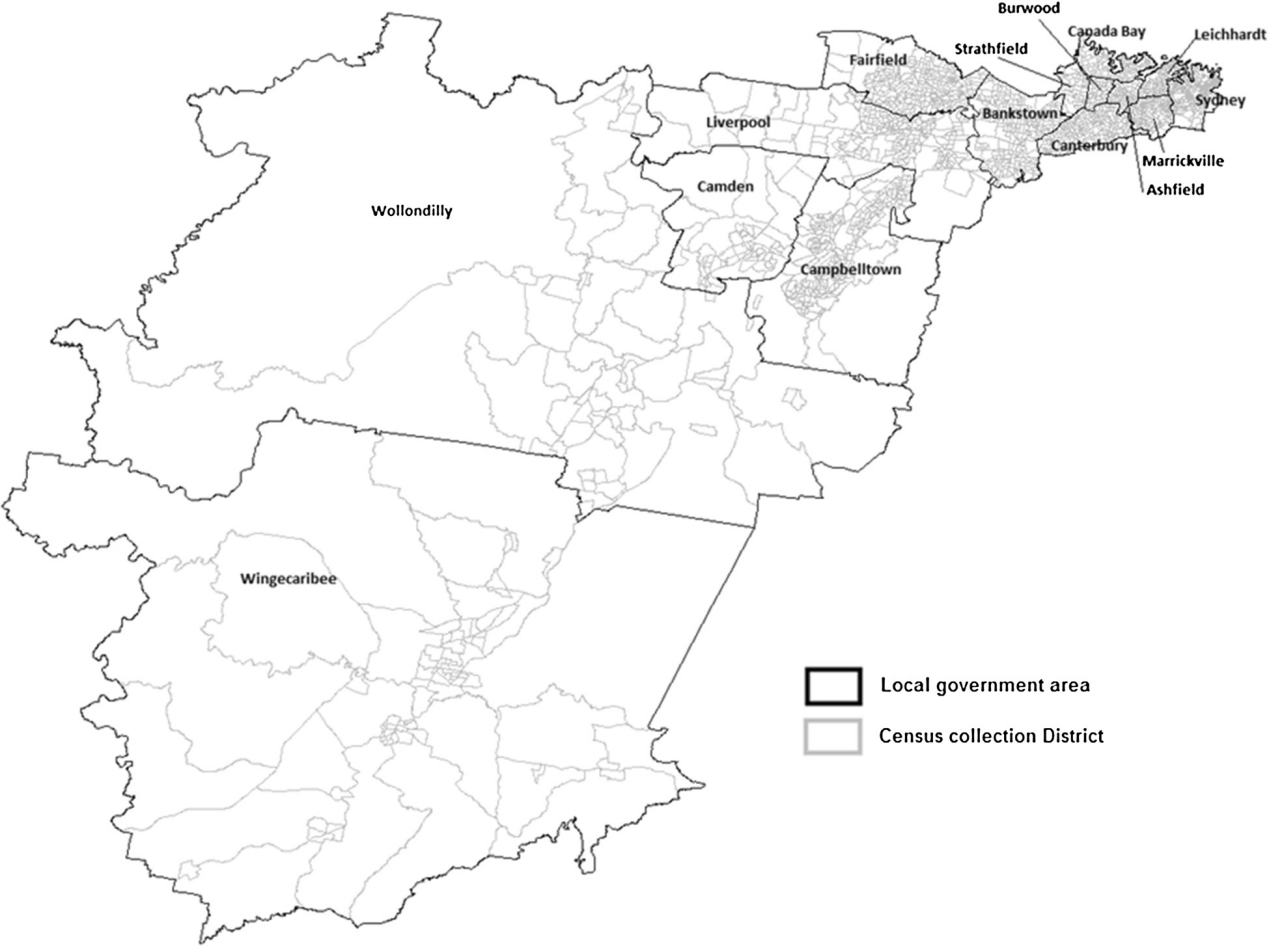
Study area of South-Western region of Metropolitan Sydney.

Figure 1 shows the study area of south-western region of metropolitan Sydney. From 1999 to 2008, there were 195,500 births in this study area. About three percent of the mothers were aged less than 20 years, 76.6% were aged between 20 and 34 years and 20.1% aged 35 years or more. Just over half of the women were born in Australia (54.6%). About 12% of women smoked during the pregnancy and 30.7% of women had their first antenatal visit ≥10 weeks of gestation. The majority of women delivered a singleton infant (97.0%). Only a small proportion of women had gestational diabetes or gestational hypertension (6.6% and 4.5% respectively (Table 1).

**Table 1:** Maternal demographic and clinical characteristics in the study area (N = 195,500)

	**n (%)**
**Age-group**	
<20 year	6431 (3.3)
20 – <35 years	149,762 (76.6)
35+ years	39,307 (20.1)
**Country of birth**	
Australian	106,769 (54.6)
Overseas	88,731 (45.4)
Index of Relative Socio-economic Disadvantage	
**(missing = 1,883)**	
Most disadvantage	64,541 (33.3)
Middle disadvantage and Least disadvantage	129078 (66.7)
**Number of babies**	
Multiple	5,886 (3.0)
Singleton	189,614 (97.0)
**Primiparous (missing = 448)**	
Yes	83,263 (42.6)
No	111,789 (57.2)
**First antenatal visit (missing = 3,049)**	
≥10 weeks	60,041 (30.7)
<10 weeks	132,410 (67.7)
**Smoking during pregnancy (missing = 1,613)**	
Yes	22,635 (11.6)
No	171,252 (87.6)

### Spatial analysis

**Figure 2:**
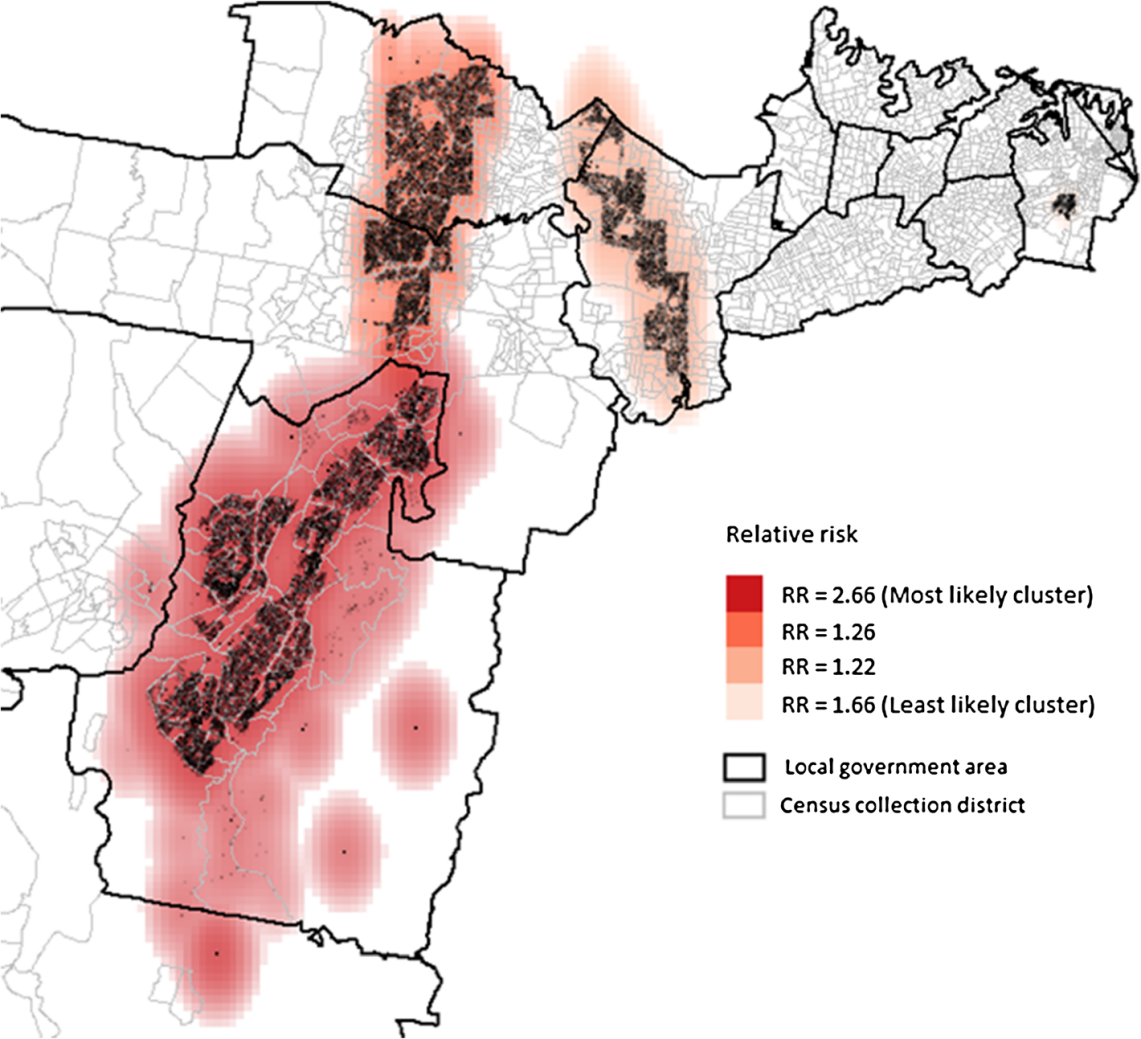
Clusters of maternal smoking during pregnancy using the maximum cluster size ≤ 20%.

**Figure 3:**
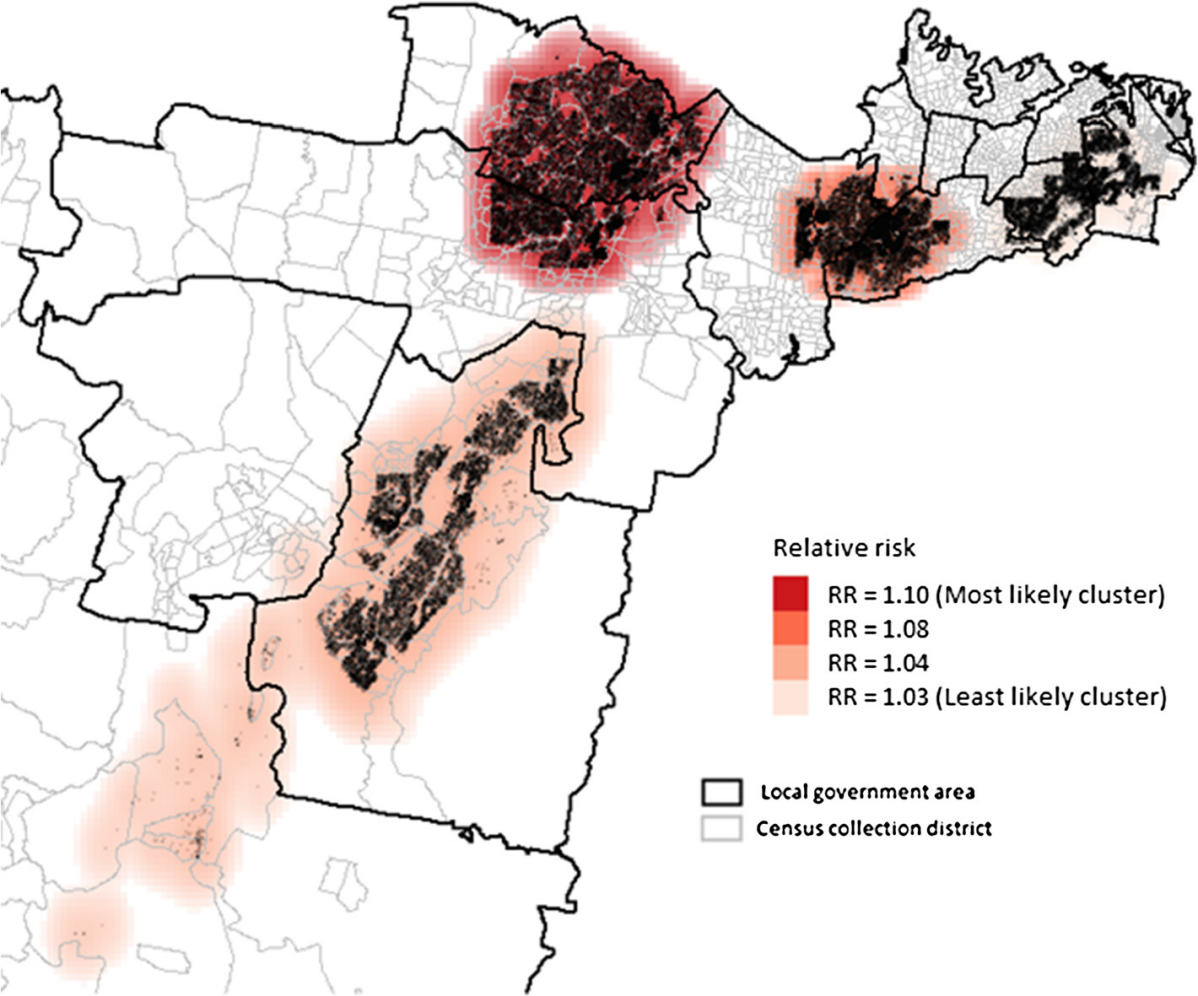
Clusters of first antenatal visit at or after 10 weeks and above using the maximum cluster size ≤ 20%.

Using the maximum spatial circular windows ≤20% of the total population, we identified a number of spatial clusters of women who smoked during pregnancy (Figure 2) and whose initial antenatal visit occurred ≥10 weeks gestation (Figure 3). These clusters are presented in order of the most likely cluster to the least likely cluster.

Four significant clusters of maternal smoking during pregnancy were generated with RRs ranging from 1.22 to 2.66. The most likely cluster covered 12.0% (n = 23,557) of all births and was mainly located in one LGA. The overall RR for this cluster was 2.66 (p < 0.01). The three secondary clusters contained 12.3% of all births.

For late first antenatal visit occurring ≥10 weeks gestation, four significant clusters were generated with RRs between 1.03 and 1.10. The most likely cluster covered 18.3% (n = 35,834) of all births in the study area and was again mainly located in one LGA (RR = 1.10, p < 0.001). The second and third were mainly located in densely populated areas of two separate LGAs and the fourth cluster covered most of a densely populated area in a LGA, with 12.5%, 10.7% and 7.8% of all births respectively.

### Characteristics of the clusters

Table 2 shows demographic characteristics for the identified clusters for maternal smoking and the first antenatal visit at or after 10 weeks gestation compared to the rest of the study area. Compared to women in the rest of the study area, women in the identified clusters for maternal smoking during pregnancy (n = 47,593 in the significant spatial clusters, 24% of all births), were aged less than 35 years (85% vs 78%; p < 0.0001) and have their first antenatal visit at or after 10 weeks (79% vs 75%, p = 0.011). The clusters also had significantly lower proportions of women who were primiparous (37% vs 45%; p < 0.0001).

**Table 2:** Demographic characteristics for significant spatial cluster compared to the remainder of the study area using GEE logistic regression

	**Smoking during pregnancy clusters n (%)**			**1st antenatal visit at ≥10 weeks clusters n (%)**		
	**Clusters**	**Remainder of the study area**	**p-value**	**Clusters**	**Remainder of the study area**	**p-value**
	**47,593**	**147,907**		**96,308**	**99,192**	
**Smoke during pregnancy**	8,767 (18.6)	11,768 (8.1)	<0.0001	11,703 (12.3)	8,832 (9.1)	0.2465
**1st antenatal visit ≥10 weeks**	36,991 (79.2)	109,491 (75.1)	0.0112	77,767 (80.8)	68,715 (71.5)	<0.0001
**Overseas-born**	19,681 (41.4)	69,050 (46.7)	0.5045	52,914 (54.9)	35,817 (36.1)	0.0623
**Maternal age (years)**						
<35	40,552 (85.2)	115,607 (78.2)	<0.0001	79,123 (82.2)	77,036 (77.7)	0.3988
≥35	7,034 (14.8)	32,273 (21.8)		17,171 (17.8)	22,136 (22.3)	
**Index of Relative Socio-economic Disadvantage**						
Most disadvantaged	20,447 (44.0)	44,094 (30.0)	0.4621	56,621 (56.4)	10,920 (11.1)	0.0014
Middle and least disadvantaged	26,078 (56.1)	103,000 (70.0)		41,437 (46.6)	87,641 (88.9)	
**Plurality**	1,386 (2.9)	4,500 (3.0)	0.1162	2,748 (2.9)	3,138 (3.2)	0.0021
**Primiparous**	17,616 (37.0)	65,647 (44.5)	<0.0001	40,000 (41.5)	43,263 (43.8)	0.8380

In the identified clusters for women who had their first antenatal care ≥10 weeks gestation, there were 96,308 (49% of all births) women in the three clusters compared to 99,192 women in the remainder of the study area. These clusters had significantly higher proportions of women who lived in the most disadvantaged areas (56% vs 11%, p < 0.0001) compared to the rest of the study area. These clusters also had lower proportions of women who had multiple births (2.9% vs 3.2%, p < 0.0001).

**Figure 4:**
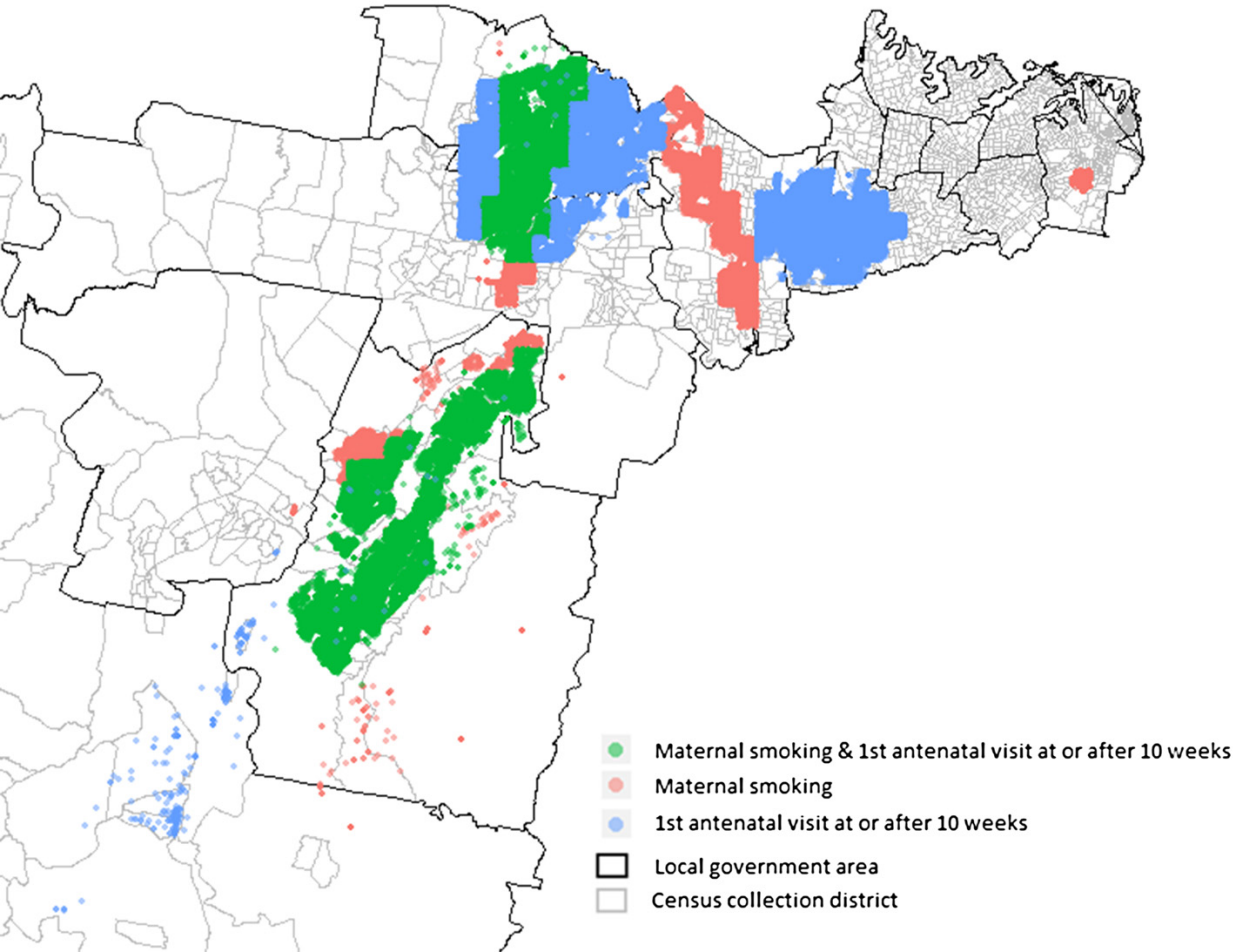
Clusters of maternal smoking overlaid with clusters of first antenatal visit at or after 10 weeks.

As the significant spatial clusters of the two risk factors overlapped, we also overlaid the clusters of maternal smoking with the clusters of first antenatal visit at or after 10 weeks of gestation to identify significant spatial clusters of both smoking during pregnancy and late antenatal visits (Figure 4). Areas around the green dots in Figure 4 indicate areas where there is a high risk for both maternal smoking and late first antenatal visit at or after 10 weeks of gestation.

**Figure 5:**
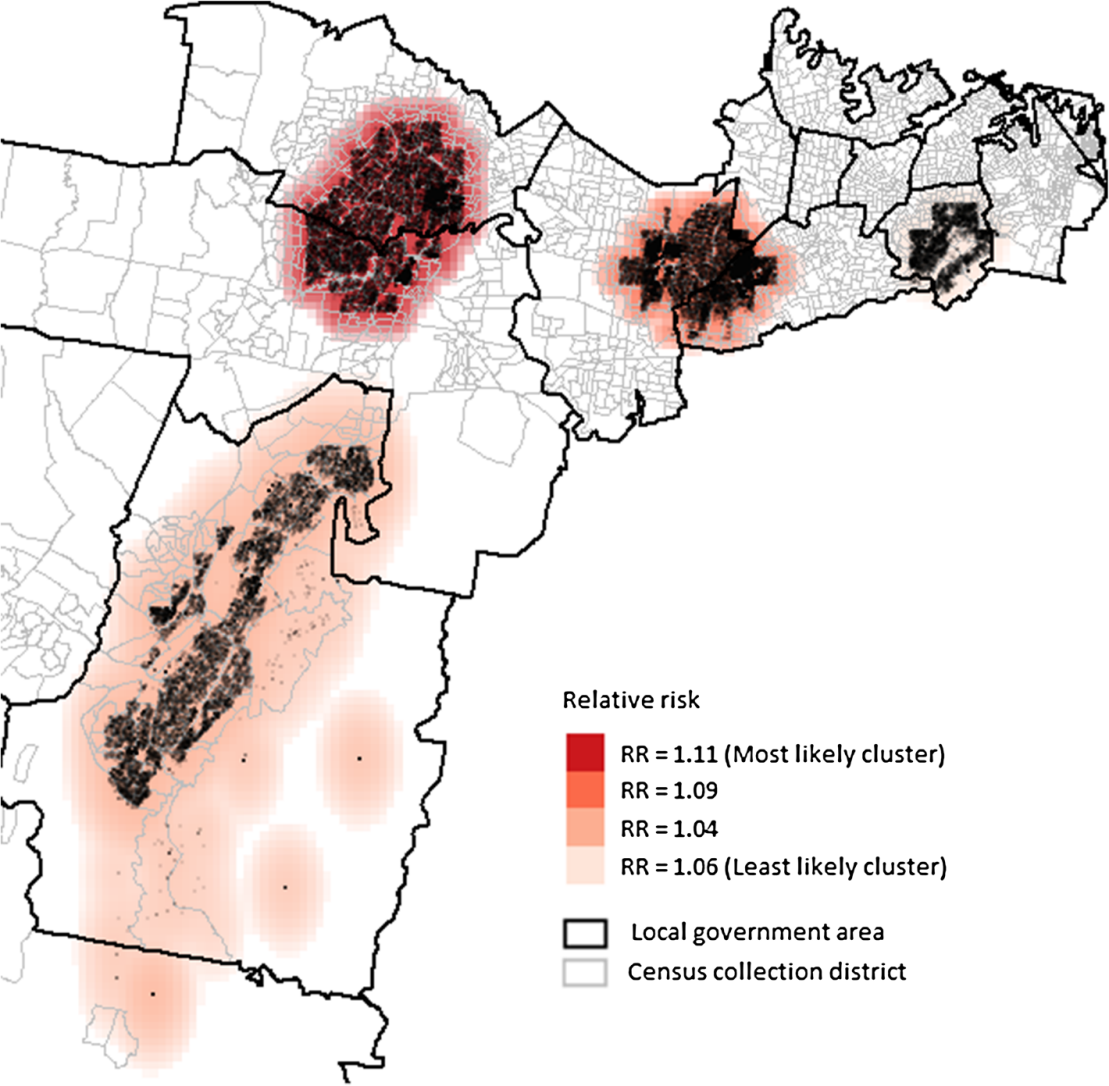
Clusters of first antenatal visit at or after 10 weeks with the maximum cluster size 10% of the total study population.

Figure 5 shows an additional spatial analysis based on a maximum circular window of 10% of the total study population. This additional analysis identified four significant spatial clusters as shown in Figure 2 when using a maximum circular window of 20% of the population. These four spatial clusters are located in the same spatial clusters identified with a maximum circular window of 20% of the study population (Figure 2), but are smaller in size. The most likely cluster in this additional analysis comprised 9.7% (n = 19,061) of all births in the study area compared to 18.3% (n = 35,834) of births based on a maximum circular window of 20% of the total study population.

## Discussion

We identified four spatial clusters of maternal smoking during pregnancy and four spatial clusters of first antenatal visit occurring at or after 10 weeks of gestation. Following identification of the clusters, we were also able to characterise pregnant women within the clusters and compare those characteristics with women living outside of those clusters. For example, in the maternal smoking during pregnancy clusters, higher proportions of mothers, were aged less than 35 years, were born in Australia, had their first antenatal visit at or after 10 weeks of gestation and a lower proportion of mothers were primiparous. In the clusters of late first antenatal visit, a higher proportion of mothers lived in the most disadvantaged areas and a lower proportion of mothers were primiparous.

SaTScan is a widely used and accepted software for geospatial analytic technique to identify and describe geographic patterns of interest in the public health literature [38-41]. Our study used both spatial statistics and descriptive statistics to describe adverse maternal-related behaviours in the south western region of metropolitan Sydney. However, it should be noted here that, although we have demonstrated the usefulness of using SatScan to identify the spatial distribution of adverse risk factors, SaTScan can also be used to identify spatial distributions favourable risk factors or health outcomes, for example, areas with low rates of smoking or certain cancers. An identified spatial cluster where the causal mechanism is not readily apparent could have merely occurred by chance [42]. Therefore, any identified clusters need to be further investigated and the results and conclusions supported by other published studies [42]. For example our finding of significant differences in risk of smoking during pregnancy across socio-demographic groups is consistent with previous studies [22,23]. Similarly, the identification of differences in risk of late antenatal care across our study area is consistent with previous studies that late antenatal care is related to mother’s age [15], area of residence [15,43] and low socio-economic status [15,16,44]. This reinforces the robustness of the results obtained from SaTScan.

Demonstrating differences in the socio-demographic factors underlying the identified clusters can assist policy makers in understanding the epidemiology of particular diseases, risk factors or health issues, and to develop and implement interventions and reorient health services. For example, SaTScan has been used to implement anti-marlarial interventions at the household level [45], to identify clusters of hypoplastic left heart and assess genetic and environmental factors contributing to hypoplastic left heart [46], and planning regional tuberculosis prevention and control strategies by identifying clusters of high incidence of tuberculosis [47].

Our study, by identifying adverse maternal-related behaviours by spatial clusters or geography, provides valuable information about the geographical disparity of adverse maternal-related behaviour. It also provides additional insights to the characteristics of the cluster that contribute to adverse perinatal outcomes. Local knowledge and understanding of the physical characteristics of the identified geographical areas (for example, availability of public transport, location of health services) and socio-demographic characteristics of the women within the clusters can assist policy makers to focus the scope of prevention/intervention programs and changes to health care delivery, thus providing more effective programs to suit individual needs and public health resources can be delivered more efficiently.

We also overlaid the clusters of maternal smoking with the clusters of first antenatal visit to identify significant spatial clusters of both smoking during pregnancy and late antenatal visits. Therefore, if there are limited resources, targeting initially only those spatial clusters with both risk factors may be a cost-effective approach to improving maternal and birth outcomes.

Public health interventions can be directed at a number of geographic levels. They can be at a national level (for example, smoking cessation campaigns using the mass media) at the one extreme to very local interventions (for example, modifying the physical characteristics of a locality) at the other extreme. Interventions at both these geographic levels can be equally valid and important. SaTScan allows us to vary the size of the scan windows and hence the number and geographic dimensions of the clusters.

Clusters generated using a larger size scan windows produce larger clusters which can help policy makers make decisions at larger geographic levels, for example, at the state of regional level. However, these large spatial clusters will cover a large area with a larger and more heterogeneous population. Conversely, clusters generated using smaller circular windows will produce smaller clusters but will contain a more homogeneous population which can help policy makers in planning more focused community interventions [48,49]. For example, Fang et al. used circular windows no more than 20% to identify hemorrhagic fever with renal syndrome clusters and smaller circular windows no more than 10% to identify possible subclusters for more efficient resource allocation for preventing hemorrhagic fever with renal syndrome [41].

The strengths of this study is the use of georeferenced address of each individual case (only 0.1% of the addresses were not be able to retrieved) which offered more geographic precision than studies based on administrative boundaries such as local government area, postal area or CCD. Using SaTScan enables us to analyse variations in any event of interest for many possible small and large geographic groups and without being restricted by administrative boundaries. In SaTScan, the shape of the scan window can be circular or elliptical. Circular windows provide good level of accuracy if the population at risk exists in circular shaped areas [29,50,51]. However, as population at risk do not exist in circular shaped areas, elliptical-shaped scan windows will provide slightly increased power for identifying non-circular shaped clusters, for example, long narrow clusters [52]. Tango and Takahashi recently proposed using flexible shaped spatial scan statistics to accommodate irregular shaped clusters [51]. Another advantage is that where point data are not available, the centroids of the smallest geographical spatial unit that are available can be used in SaTScan [52]. Lastly, SaTScan is also capable of performing space-time scan statistics to identify clusters existing in both space and time [34].

However, there are also a number of limitations of using SaTScan to identify spatial clusters. For those wishing to explore modelling based approaches, SaTScan only implements scan statistic methods. Also, whilst it can identify clusters, it cannot explain why the variations in the risks of the events of interest exist. SaTScan also lacks an interface to other graphing, mapping and statistical packages. Another limitation of SaTScan is that it does not provide guidance for choosing the maximum size of the scan windows [40]. We were also unable to link multiple births to a mother and therefore could not account for this in our analyses. This could underestimate the standard error and overestimate the significance of socio-demographic characteristics. Further, we were not able to include all potential predictors or confounders of adverse maternal –related behaviours in our models as they were not available in our dataset. Nevertheless, geospatial analytical techniques are useful tools for identifying geographic areas for intervention. However, the usefulness of these geospatial techniques is only as good as the quality of the data that are available. Finally, although not a limitation of the study, an important point to bear in mind is that due to the large number of women in our study, small differences between women in the spatial clusters and women outside of the spatial clusters were likely to be detected as statistically significant in the descriptive analysis. The epidemiological and clinical importance of such small differences should also be considered in decision making rather than simply relying on statistical significance in informing decisions.

## Conclusion

This study provides a first attempt, as far as we are aware, to visually and quantitatively identify and describe the spatial characteristics of ante-natal risk factors. We have demonstrated that using existing georeferenced routinely collected health data, GIS and geospatial techniques can be useful tools to assist policy makers to implement targeted interventions in smaller geographic areas and which will also ensure that limited resources are used efficiently.

## Competing interests

The authors declare that they have no competing interests.

## Authors’ contributions

SC conducted the literature search, the analyses and drafted the manuscript. MN assisted with the analyses and provided expert advice on geospatial statistics. BJ, EH and JE conceptualised the ideas for this manuscript. All authors contributed to, and approved, the final manuscript.
